# Arthroscopic internal drainage of popliteal cysts with cyst wall resection in pediatric patients

**DOI:** 10.12669/pjms.38.8.5354

**Published:** 2022

**Authors:** Junxu Zhu, Dulei Xiang, Shen Yang, Liangbi Xiang, Xinwei Liu

**Affiliations:** 1 Junxu Zhu, The General Hospital of North Theater Command Training Base, Jinzhou Medical University Graduate, Shenyang 110016 Liaoning, People’s Republic of China; Department of Orthopaedics, Xiangyang Hospital of Traditional Chinese Medicine, Xiangyang 441000 Hubei, People’s Republic of China; 2 Dulei Xiang, Department of Orthopaedics, The General Hospital of Northern Theater Command, 83 Wenhua Road, Shenyang 110016 Liaoning, People’s Republic of China; 3 Shen Yang, Department of Orthopaedics, The General Hospital of Northern Theater Command, 83 Wenhua Road, Shenyang 110016 Liaoning, People’s Republic of China; 4 Liangbi Xiang, Department of Orthopaedics, The General Hospital of Northern Theater Command, 83 Wenhua Road, Shenyang 110016 Liaoning, People’s Republic of China; 5 Xinwei Liu, Department of Orthopaedics, The General Hospital of Northern Theater Command, 83 Wenhua Road, Shenyang 110016 Liaoning, People’s Republic of China

**Keywords:** Arthroscopy, Knee joint, Popliteal cyst

## Abstract

**Objectives:**

To evaluate the efficacy of arthroscopic internal drainage (AID) and cyst wall resection (CWR) in children with popliteal cysts.

**Methods:**

This study included 16 pediatric patients with popliteal cysts and received arthroscopy using the double posteromedial (PM) portal system during June 2020 and June 2021 at The General Hospital of Northern Theater Command. Among these pediatric patients, 14 were males and two were females, with the mean age of nine years (range: 7-12 years). The left knee was affected in 12 cases, while the right knee was involved in the rest four cases. All patients underwent MR imaging before the procedure to assess whether there was intra-articular trauma and whether the popliteal cyst communicated with the knee-joint cavity. The MRI results showed that each patient had a simple popliteal cyst that involved a single knee joint without intra-articular trauma, which was classified as Grade-1 (n=3), Grade-2 (n =10) or Grade-3 (n =3) according to the Rauschning and Lindgren grading of knee joint symptoms. Arthroscopy was performed through anterolateral (AL) and PM portals to the knee joint for AID plus CWR, and the surgical outcomes were evaluated based on the Rauschning and Lindgren criteria.

**Results:**

No major vascular or nerve injury occurred during the operation. Postoperative complications such as wound infection and lower-extremity deep venous thrombosis were not recorded in these patients. Complications involving the saphenous nerve or the great saphenous vein or pseudocyst formation were not observed during the follow-up period. All patients completed the follow-up ranging from 3-12 months and were identified to have grade-0 (n=15) and grade-1 (n=1) popliteal cysts based on the Rauschning and Lindgren criteria, indicating significant improvement compared with the preoperative levels (all p<0.05). Moreover, no recurrence was recorded after operation.

**Conclusion:**

AID plus CWR is a minimally invasive and safe approach for pediatric patients with popliteal cysts to promote postoperative recovery and reduce the recurrence rate.

## INTRODUCTION

A popliteal cyst is a commonly seen condition of the knee joint. This concept became widely known among surgeons since it was first described by Robert Adams in 1840. Popliteal cysts are also known as Baker’s cysts to honor the British surgeon William Morant Baker, who gave an elaborate description of the disease in 1877.^1^ Popliteal cysts are either primary or secondary. Adults are often found with secondary popliteal cysts accompanied by arthropathies such as meniscus injury, degeneration of joint, and osteoarthritis. Popliteal cysts in adults usually require an active probe into the affected joint and appropriate treatment of intra-articular lesions. Therefore, the anterior and posterior approaches are adopted in most cases to treat popliteal cysts in adults. Causes of popliteal cysts in children are different from those in adults. Most pediatric patients have primary popliteal cysts, and intra-articular trauma is rarely seen in these cases.^2^ However, since intra-articular lesions cannot be completely ruled out by preoperative screening, the 16 pediatric patients admitted by our hospital due to popliteal cysts between June 2020 and June 2021 were treated with arthroscopic internal drainage (AID) plus cyst wall resection (CWR) via anterolateral (AL) and posteromedial (PM) portals, which is reported as follows.

Our objective was to evaluate the efficacy of arthroscopic internal drainage (AID) and cyst wall resection (CWR) in children with popliteal cysts.

## METHODS

**Fig.1:**
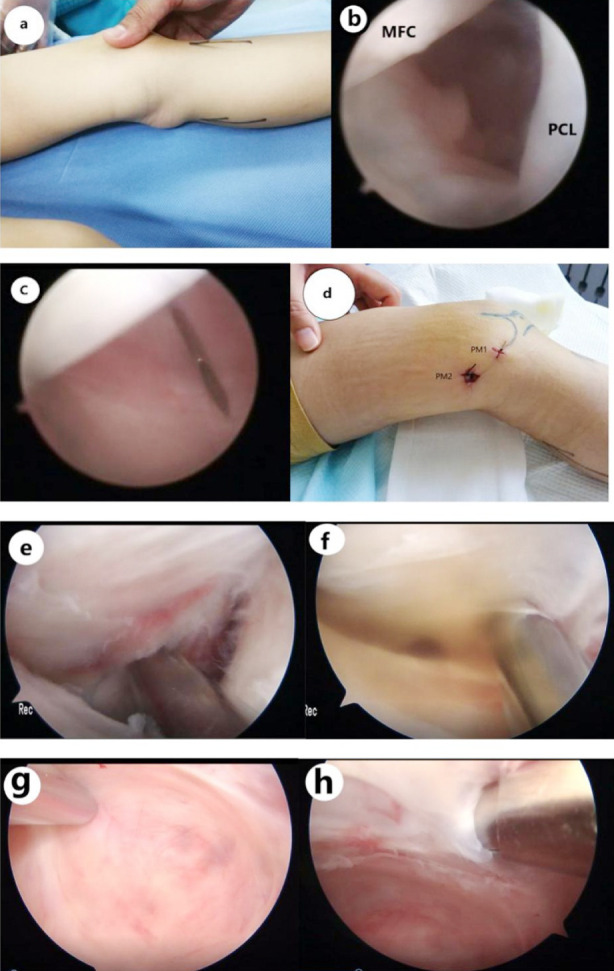
Preoperative examination showed a mass in the medial popliteal fossa, which was remarkable in knee extension and became unremarkable in knee flexion (Foucher’s sign) (a). Arthroscope was inserted into the PM compartment (b) from between the PCL and medial epicondyle of the femur; a lumbar puncture needle was inserted into the PM space to create the first PM portal (PM1) (c-d). A shaver was inserted from PM1 for expansion of the valvular structure to reveal the medial head of the gastrocnemius (e). After probing the cyst valvular opening, there was a massive flow of bright yellow liquid into the knee-joint cavity (f). An exchange rod was inserted into the cyst lumen from PM1 to push the arthroscope against the cyst wall. The second PM portal (PM2) was created behind PM1 using a lumbar puncture needle, and a shaver was inserted for expansion of the cyst valvular opening to expose and remove the inner cyst wall (g) with the shaver in low-suction condition (h).

This study included 16 patients who were admitted due to a cystic mass arising in the popliteal fossa with or without pain during June 2020 and June 2021 at The General Hospital of Northern Theater Command(Fig.1a), including 14 males and two females at the age of seven to tweleve years old, with the mean age of nine years. The left knee was affected in 12 cases, while the right knee was involved in the rest four cases. The course of disease ranged from four to 12 months. MR imaging of the affected knee was performed before surgery to confirm whether there was intra-articular trauma or communication between the cyst and the knee-joint cavity. Patients who had a history of surgical treatment of the affected knee or failed to complete the follow-up were excluded from the study. The Rauschning and Lindgren criteria^3^ are briefly described as follows: Grade-0: no swelling, pain or limitation of range of motion; Grade-1: slight swelling and/or discomfort of the popliteal fossa after strenuous exercise, and negligible limitation of range of motion; Grade-2: swelling and pain following moderate exertion, and limitation of range of motion<20 degrees; Grade-3: swelling and pain even at rest, and limitation of range of motion>20 degrees. According to the Rauschning and Lindgren criteria, participants in this study had Grade-1 (n=3), Grade-2 (n=10) and Grade-3 (n=3) cysts. The study was approved by the Institutional Ethics Committee of The General Hospital of Northern Theater Commandon on September 20, 2019 (No.[2019]087), and written informed consent was obtained from all participants.

### Surgical Procedures:

The patient was placed in a prostrate position. General anesthesia was performed (as an alternative to continuous epidural anesthesia because pediatric patients tended to show poor compliance and have a bad experience of surgery). Following that, a tourniquet was positioned at the root of the thigh, and the patient was prepped and draped in the usual sterile fashion. The knee joint was examined routinely via the AL portal. If the patient was confirmed to be free of any intra-articular lesions such as meniscus tears, synovitis and free bodies - which were discovered in none of the patients in this study, the arthroscope was inserted into the PM compartment (Fig.1b) from between the posterior cruciate ligament (PCL) and the medial epicondyle of the femur. The PM compartment could be observed with the knee at 90 degrees of flexion, and a lumbar puncture needle was inserted into the PM space (Fig.1c) to create the first PM portal (PM1) (Fig.1d). A shaver was inserted for the removal of folds of the synovial membrane to identify and position the medial head of the gastrocnemius. Generally, the cyst valvular opening lies on the PM side of the medial head of the gastrocnemius, sometimes covered by the synovium.^1^,^4^ The valvular structure was expanded by inserting a shaver from PM1 to reveal the medial head of the gastrocnemius (Fig.1e). The cyst valvular opening was successfully captured when there was a massive flow of bright yellow liquid into the knee-joint cavity (Fig.1f). An exchange rod was inserted into the cyst lumen from PM1. Then, the arthroscope was pushed by the exchange rod against the cyst wall. The second PM portal (PM2) was created 2cm away from behind PM1 using a lumbar puncture needle, and a shaver was inserted for expansion of the cyst valvular opening to expose the inner cyst wall (Fig.1g), which was removed using a shaver in low-suction condition (Fig.1h). If multilocular cyst formation arose from fibrous separation in the inner cyst wall, the fibrous separation should be removed without affecting the peripheral nerves and blood vessels. Subsequently, the arthroscope was switched to PM2 using the exchange rod, with PM1 as an operating portal. Then, the residual cyst wall was resected from between the semimembranosus bursa and the medial head of the gastrocnemius. After AID and CWR, the knee-joint cavity was rinsed thoroughly. A drainage tube was placed in the cavity after the stoppage of bleeding and washing, followed by suturing and pressure dressing of the incision with sterilized dressing.

### Postoperative treatment:

After surgery, the affected limb was dressed with pressure bandages. The patient was administered with medications to relieve swelling and pain in combination with conventional anti-infection therapy for two days. All patients were commenced on the same regimen for rehabilitation following surgery. The affected knee was kept in extension as often as possible after surgery and treated with cold compress therapy within the first 48 hour. Off-bed activities were allowed the next day after the drainage tube was removed, and the patient was instructed to participate in quadriceps contraction and ankle pumping exercises. Discharge was approved if no abnormality was found after three days of observation following surgery, and the patient might visit our outpatient clinic to change dressings.

### Data Analysis:

The statistical software SPSS 22.0 was used for data analysis. Comparison of ranked and grouped data was examined by the two-sample signed-rank test (i.e., the Wilcoxon two-sample test), with the significance level α at 0.05.

## RESULTS

**Fig.2:**
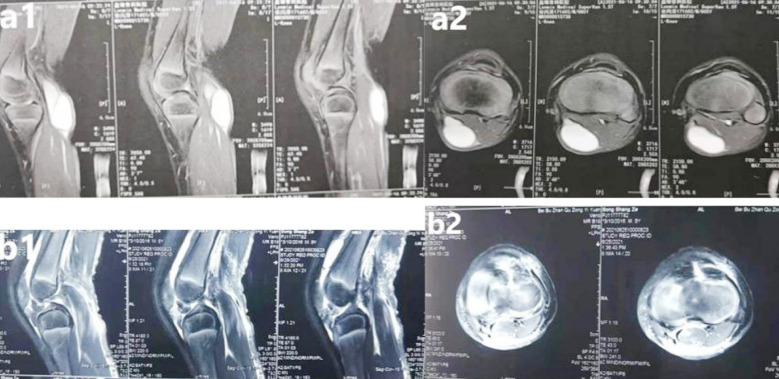
Popliteal cyst was visible in the PM side of the knee on the preoperative sagittal and axial images (a1-2). Shadow of popliteal cyst was not found on the postoperative sagittal and axial images (b1-2).

MR imaging of the affected knee was performed after surgery for comparison with the preoperative condition, and the results demonstrated removal of the valvular structure and disappearance of the cyst (Fig.2). All patients completed follow-ups one, three, six and tweleve months after surgery. The mean follow-up period was nine months (range: three to tweleve months), during which the therapeutic effect was assessed with the Rauschning and Lindgren criteria. No major vascular or nerve injury occurred during the operation. Postoperative complications such as wound infection and lower-extremity deep venous thrombosis were not recorded in these patients. Complications involving the saphenous nerve or the great saphenous vein or pseudocyst formation were not observed during the follow-up period. Differences between the pre- and post-operative scores based on the Rauschning and Lindgren criteria were statistically significant (p<0.05), with the postoperative score markedly higher than the preoperative score (Table-I).

**Table-I:** Comparison of pre- and post-operative scores based on the Rauschning and Lindgren criteria (n=16).

Time of Observation	Grade 0	Grade-1	Grade-2	Grade-3
Preoperative	0	3	10	3
Postoperative	15	1	0	0

***Note:*** p<0.05 indicated a difference of statistical significance between the pre- and post-operative scores.

## DISCUSSION

Popliteal cysts, also known as Baker’s cysts, were first described and identified by Robert Adams in 1840 and William Morant Baker’s in 1877.^1^ Despite the mixed opinions on the pathogenesis and treatment methods, it is generally accepted that there are two types of popliteal cysts, namely primary and secondary cysts. Primary popliteal cysts mostly occur in children, and the pathogenesis is still unclear. In pediatric or adolescent patients, popliteal cysts are usually formed by the medial head of the gastrocnemius and the semimembranosus bursa, without communication with the knee-joint cavity (except those induced by joint injury or infection).^5^ Secondary popliteal cysts are mostly found in adult patients following certain joint diseases, such as rheumatoid arthritis, meniscus injury, osteoarthritis, and nonspecific synovitis. Currently, it is extensively accepted that popliteal cysts develop in association with a unidirectional valvular mechanism, that is, when an effusion accumulates due to certain knee-joint conditions, the intra-articular pressure of the knee increases, leading synovial fluid to the gastrocnemius-semimembranosus bursa (GSB) by passing through the transversal fissure-like structure, which contributes to formation and persistence of cysts because the flow cannot be reversed.^6^ Moreover, pressure from the cyst and joint disease can give rise to clinical symptoms of the knee. In 2012, Chinese scholars Zhu M et al.^6^ reported that of 25 autopsy cases, 10 specimens demonstrated that GSB communicated with the synovial capsule of the knee, while the rest 15 specimens suggested no communication between the two structures but a weak spot in the PM ankle associated with the synovial capsule of the knee. Synovial fluid flows into GSB when the intra-articular pressure of the knee increases to a given level.

Related symptoms are usually associated with the size of the cyst, including pain, distending pressure and stiffness of the posterior or PM knee joint.^7^ Bryan et al.^8^ pointed out in a study on 38 patients with popliteal cysts that the most common symptoms included distending pressure in the popliteal fossa (76%) and pain in the PM knee joint (32%). During the physical examination, a round, smooth, resilient bump with a wave-like feeling could be detected upon palpation in the middle or on the lateral side of the posterior popliteal fossa.^9^ Patients may experience hyperextension pain, and the pain level is strongly associated with the range of motion. A larger cyst means a greater limitation in knee extension and flexion, especially after physical activities or overwork. The cyst tends to be firm in hyperextension and soft when the knee is flexed, which is defined as Foucher’s sign. During knee hyperextension, intra-articular pressure of the knee increases as the medial head of the gastrocnemius and semimembranosus bursa approximate each other due to cyst compression. This sign can help differentiate Baker’s cysts from other masses in the popliteal fossa, such as hemangioma, neurilemmosarcoma, and tumors independent of joint motion.^10^ MR imaging is currently a proven technique for the diagnosis of masses in the knee joint as the “gold standard” for the assessment of lesions of knee joint.^11^ Popliteal cysts in knee MRI are usually manifested by uniform low signal intensity on T1WI, uniform high signal intensity on T2WI and a beak-shaped communication port between the knee-joint cavity and the cystic lesion. MRI offers superior contrast resolution for soft tissues to accurately visualize the cystic lesion and the relationships between the cystic opening and the articular cavity as well as other peripheral structures. Moreover, it outperforms other imaging techniques in simultaneous visualization of other lesions coexisting with the cyst, such as meniscus tears, cartilage injury, ligament injury, which is of significant value in deciding surgical treatment plan and prognosis assessment. If a popliteal cyst ruptures or leaks fluid, a high signal of edema can be observed in the peripheral soft tissues on MRI.

Considering the nature of popliteal cysts in pediatric patients, the specific cause of the disease is still unclear. Unlike secondary popliteal cysts in adult patients, primary popliteal cysts in pediatric patients show no lesions in the affected knee joint. Based on the unidirectional valvular mechanism, it is critical to reestablish the bidirectional connection between the knee-joint cavity and the cyst^12^ for successful treatment. As to the communication port, Li et al.^13^ and Kp et al.^14^ considered it unnecessary to suture the communication port as a satisfactory therapeutic effect was achieved by expanding the communication port by about 5 mm to switch from a unidirectional flow to a bidirectional flow. Recently, AID has been extensively recognized as a treatment strategy for patients with popliteal cysts.^15^-^19^ However, AID combined with treatment of intra-articular lesions without CWR remains controversial as this may lead to the recurrence of the cyst. Zhang et al.^20^ discovered that the popliteal cyst wall was histologically comprised of thickened tissues of glassy degeneration, without any synovial cells. Therefore, they considered it unnecessary to remove the cyst wall completely because it was essentially a fluid-filled sac that did not produce synovial fluid. Li et al.^13^ reported that the recurrence rate had no clear association with the cyst wall but with the unidirectional valvular mechanism of the PM articular capsule and other causative factors. Zhou et al.^21^ suggested in a Meta analysis of published works on surgical treatment of popliteal cysts between 1980 and July 2015 that arthroscopy was an ideal option to treat intra-articular lesions, expand the communication port and resect the cyst wall. Su C et al.^22^ reported in a recent study that popliteal cysts disappeared completely and shrunk in 81.8% and 18.2% of all patients when combining AID with CWR, and the cyst survival rate was 0%, which indicated a further decline in the recurrence rate compared with arthroscopic decompression of simple intra-articular lesions without giving rise to such intra-operative complications as nerve and vascular injury mentioned in other published studies. This finding was proven by this study. Compared with conventional AID, it took more time to perform AID plus CWR because of the establishment of additional PM portals and a shift of position. Yet, the study^22^ also demonstrated that the operation time was largely dependent on the surgeon’s experience and the difficulty of CWR. The learning curve (5-10 cases) based on limited experience and sensations exhibited observable improvement. After surgery, the affected limb was dressed with pressure bandages, placed in extension and treated with cold compress therapy; in the meantime, quadriceps contraction and ankle pumping exercises were required to reduce the recurrence rate.

### Limitations of study:

The sample size was small. Further studies are needed to determine the optimal treatment option for patients with popliteal cysts in the lateral popliteal fossa and surrounded by blood vessels to avoid complications associated with intra-operative nerve and vascular injury.

## CONCLUSIONS

Considering the self-limiting nature of primary popliteal cysts in pediatric patients, no special treatment is needed in most cases.^23^ Despite all that, we still find it necessary to treat symptomatic popliteal cysts with a pertinent regimen. For pediatric patients with popliteal cysts, AID plus CWR is a minimally invasive and safe treatment option that features desired clinical efficacy, fast recovery, low recurrence rate and complete cyst removal.

### Authors’ Contributions:

**JZ & DX:** Designed this study, prepared this manuscript, are responsible and accountable for the accuracy and integrity of the work.

**XL:** Collected and analyzed clinical data.

**SY &** **LX:** Data analysis**,** Significantly revised this manuscript.
